# Bilingual Contexts Modulate the Inhibitory Control Network

**DOI:** 10.3389/fpsyg.2018.00395

**Published:** 2018-03-27

**Authors:** Jing Yang, Jianqiao Ye, Ruiming Wang, Ke Zhou, Yan Jing Wu

**Affiliations:** ^1^Bilingual Cognition and Development Lab, Center for Linguistics and Applied Linguistics, Guangdong University of Foreign Studies, Guangzhou, China; ^2^Guangdong Provincial Key Laboratory of Mental Health and Cognitive Science, Center for Studies of Psychological Application, School of Psychology, South China Normal University, Guangzhou, China; ^3^College of Psychology and Sociology, Shenzhen University, Shenzhen, China; ^4^Shenzhen Key Laboratory of Affective and Social Cognitive Science, Shenzhen University, Shenzhen, China; ^5^Faculty of Foreign Languages, Ningbo University, Ningbo, China

**Keywords:** bilingualism, inhibitory control, dual-language contexts, fMRI, effective connectivity

## Abstract

The present functional magnetic resonance imaging (fMRI) study investigated influences of language contexts on inhibitory control and the underlying neural processes. Thirty Cantonese–Mandarin–English trilingual speakers, who were highly proficient in Cantonese (L1) and Mandarin (L2), and moderately proficient in English (L3), performed a picture-naming task in three dual-language contexts (L1-L2, L2-L3, and L1-L3). After each of the three naming tasks, participants performed a flanker task, measuring contextual effects on the inhibitory control system. Behavioral results showed a typical flanker effect in the L2-L3 and L1-L3 condition, but not in the L1-L2 condition, which indicates contextual facilitation on inhibitory control performance by the L1-L2 context. Whole brain analysis of the fMRI data acquired during the flanker tasks showed more neural activations in the right prefrontal cortex and subcortical areas in the L2-L3 and L1-L3 condition on one hand as compared to the L1-L2 condition on the other hand, suggesting greater involvement of the cognitive control areas when participants were performing the flanker task in L2-L3 and L1-L3 contexts. Effective connectivity analyses displayed a cortical-subcortical-cerebellar circuitry for inhibitory control in the trilinguals. However, contrary to the right-lateralized network in the L1-L2 condition, functional networks for inhibitory control in the L2-L3 and L1-L3 condition are less integrated and more left-lateralized. These findings provide a novel perspective for investigating the interaction between bilingualism (multilingualism) and inhibitory control by demonstrating instant behavioral effects and neural plasticity as a function of changes in global language contexts.

## Introduction

Bilingualism is a form of “mental juggler” (Kroll and Bialystok, 2013), as speaking one language often involves simultaneous access to the non-target language in the brain (Dijkstra and Van Heuven, 1998; Green, 1998; Bialystok, 2007; Thierry and Wu, 2007; Wu and Thierry, 2010, 2017; De Groot, 2012). Therefore, for bilingual speakers, managing two languages requires constantly selecting words in the intended language and suppressing activations of the non-target language, a routine that necessitates the engagement of inhibitory control. As a result, the experience of using multiple languages might enhance bilinguals’ performance in non-linguistic domains. Previous studies have shown that bilinguals are less distracted, as compared to monolinguals, when performing inhibitory control tasks, including the Simon task (Bialystok et al., 2004; Martin-Rhee and Bialystok, 2008), the Stroop task (Bialystok et al., 2008), and the flanker task (Costa et al., 2008). Moreover, interpreting training has been shown to improve inhibitory control processes (Dong and Zhong, 2017).

The past decade has witnessed a dramatic increase in the use of neuroimaging techniques such as functional magnetic resonance imaging (fMRI) to study the neural system of bilingual language control and the effects of second language acquisition on inhibitory control (e.g., Bialystok et al., 2005; Luk et al., 2010; Weissberger et al., 2015). Abutalebi and Green (2007) proposed a brain network for language control during bilingual speech production. The network includes the left prefrontal cortex, anterior cingulate cortex (ACC), basal ganglia, and inferior parietal lobule. Abutalebi and Green’s (2007) hypothesis is that this neural network is dedicated to the selection and temporal sequencing of language representations during bilingual word production, and the pipeline works in the following order: The left basal-ganglia and ACC modulate the neural activity levels in the left prefrontal cortex, which influences neural activity in the inferior parietal cortex. Each of these areas has implications in distinct cognitive processes: The prefrontal cortex inhibits the non-target language and corrects errors; the ACC monitors conflicts and detects errors; the basal ganglia, especially the caudate nuclei, supervises the language selection and lexical access; the inferior parietal lobule, as a key region for working memory, serves a goal maintenance function. Abutalebi and Green (2008) further clarified distinct contributions of the left and right supramarginal gyri (SMG) in the inferior parietal lobules: The Left SMG is responsible for bias selection away from the language not in use; on the contrary, the right SMG is responsible for bias selection toward the language in use. This neural network for bilingual language control has been testified in a number of studies. In an fMRI study on German–Italian–English trilinguals, Abutalebi et al. (2013a,b) showed that language-switching directions influenced brain activation levels in the caudate nuclei, while activation levels of the supplementary motor area (SMA)/ACC did not vary as the function of language proficiency, which suggests a domain-general role for SMA/ACC in control tasks. Using meta-analysis approaches, Luk et al. (2012) showed that 10 neuroimaging studies involving language switching reported significant and reliable neural responses in the following brain regions: The left inferior frontal gyrus (IFG), left middle temporal gyrus (MTG), left middle frontal gyrus (MFG), right precentral gyrus, right superior temporal gyrus (STG), midline pre-supplementary motor area, and bilateral caudate nuclei. Taken together, it is clear that the prefrontal cortex and caudate nuclei are highly involved in the regulation of bilingual speeches. These brain areas have also been reported in studies of non-linguistic cognitive tasks (e.g., Luk et al., 2010). However, how these areas are connected with one another as part of the control network remains unclear.

Taking the advantage of the functional connectivity approach, researchers have attempted to identify the interactions between the language control network and the cognitive control network during L2 acquisition. For example, Ghazi Saidi et al. (2013) in an L2 vocabulary training study showed that the language processing network and cognitive control network were highly integrated at the initial stage of vocabulary learning, but as the learning proceeded and the vocabularies are being consolidated, this integration decreased. Grant et al. (2015) expanded this line of research. Instead of lab-based short-term vocabulary training, they studied neural adaptations in the development of L2 processing by examining a group of classroom Spanish L2 learners who were native English speakers over the course of one academic year. Their results show that with increased L2 experience, the overall activations in the control areas such as the ACC decreases while its connectivities with semantically related regions such as the MTG increases. The authors claim that the ability to utilize cognitive control mechanisms to regulate access to the L2 representations is a more critical issue in the beginning, relative to the latter stage, of L2 acquisition. Taken together, these studies suggest an important role of the cognitive control network in early L2 acquisition.

One possibility is that the high demand and long-term practice of language control, which involves inhibitory control, during in L2 acquisition, that allows bilinguals outperforming monolinguals in several cognitive control tasks. However, participants’ background variables, such as socioeconomic status and ethnic origins (Sabbagh et al., 2006; Morton and Harper, 2007; Li et al., 2013), also seem to matter for the cognitive advantage in bilinguals. It is also possible that language processing contexts account for some of the variances (e.g., Wu and Thierry, 2013). Studies of neural plasticity on high temporal scales (Fields, 2005; Delekate et al., 2011; Bercury and Macklin, 2015) support the notion that different global language contexts (single or dual-language contexts) may lead to distinguished neural activation patterns during target word selection (Green, 2011). Green and Abutalebi (2013) proposed an adaptive control hypothesis: Language control processes adapt to the recurrent demands of the interactional context. For example, in a dual-language context, in which both languages are used (but to different speakers), language processing engages the control network comprising bilaterally inferior frontal and parietal cortices, the ACC/pre-SMA, basal ganglia, and thalamus (Abutalebi and Green, 2016). In a dense code-switching context, however, speakers routinely interleave their languages in the course of a single utterance and adapt words from one of language in the context of the other language. The neural network of language control would rely more on a cerebellar-prefrontal connection as compared to the dual-language context because, in a dense code-switching context, language control involves higher demands for opportunistic planning (Abutalebi and Green, 2016).

Although the adaptive control hypothesis is a recent theory on the neural mechanisms of bilingual control, there has been increasing interest in the influence of language contexts on non-linguistic executive functions, such as inhibitory control. In a study using event-related potentials (ERPs), Wu and Thierry (2013) examined effects of immediate changes in language processing contexts on executive function in a group of early Welsh–English bilinguals. The cognitive control performance of these participants was measured using a modified version of the classic flanker task, in which participants were instructed to press a button to indicate the direction of an arrow presented within an array of flankers (arrows pointing to the same or the opposite direction). Critically, a word is presented before the flanker trial to implicitly prime a language context. The contextual words were either in Welsh (L1), English (L2), or both languages in separated blocks. The results showed higher accuracy rates when bilingual participants performed incongruent trials of the flanker task in the dual-language context as compared to single-language contexts. The P300 amplitude was also reduced in the dual-language, as compared to the single-language context, indicating less flanker interference effect in the Welsh–English context. Therefore, the authors claimed that changes in language processing contexts could modulate non-linguistic cognitive control in bilinguals.

In a further exploration, Liu et al. (2016) examined the effect of language contexts on cognitive control in a group of Chinese–English bilinguals. Unlike the highly proficient Welsh–English bilinguals in Wu and Thierry (2013), participants in Liu et al. (2016) were native speakers of Chinese who have a moderate level of proficiency in English. All participants performed an antisaccade task, which measures response inhibition (or response suppression), interference inhibition (or inhibitory control), and task switching, three key subcomponents of executive functions (Bialystok et al., 2006). Response suppression refers to the ability to withhold an inappropriate response (e.g., triggered by a habitual cue), as is most classically established in the go/no-go paradigm. Inhibitory control refers to the process when multiple sources of information (e.g., the printing color and the word meaning in the classic Stroop paradigm) are competing for attention which needs to be drawn to the target attribute of the stimulus. Task switching refers to the ability to alter between two tasks that require different cognitive processes and responses. The critical difference between response suppression and inhibitory control is that the former taps onto the process of response execution, whereas the latter mainly measures the control of selective attention. In Liu et al. (2016), Chinese–English participants performed an antisaccade task in the pre-test and then complete a cued digit-naming task involving both Chinese and English. Following the naming task, the participants performed the same antisaccade task again in the post-test. The results showed that the bilingual naming task enhanced response suppression, impeded the inhibitory control, and made no influence on the performance of task switching. Therefore, the authors suggest that moderate proficient bilinguals may rely heavily on response suppression when making speech production in two languages. As a consequence, the bilingual naming task improved their performance in the antisaccade task. Meanwhile, because of the limited cognitive resources and more involvement of response suppression, inhibitory control might have been allocated with less cognitive resources when moderate proficient bilinguals name digits using alternating languages, explaining the decreased performance in inhibitory control. Task switching involves a different mechanism from response suppression and inhibitory control and was not influenced by the bilingual context.

To reconcile discrepancies in previous studies, the present study explores the effect of language contexts on the neurocognitive mechanism of inhibitory control in a group of Cantonese–Mandarin–English trilinguals, who were highly proficient in Cantonese (L1)^1^ and Mandarin (L2), and moderate proficient in English (L3). One possibility is that the discrepancies between Wu and Thierry (2013) and Liu et al. (2016) are not necessarily contradictory; they might arise as a result of differences in the participants’ language background. The Welsh-English bilingual participants in Wu and Thierry (2013) were highly proficient in both languages; in contrast, the Chinese–English bilinguals in Liu et al. (2016) were intermediate learners of English. Bilinguals with high and low levels of L2 proficiency might adopt different processing strategies during speech production and, therefore, have incomparable implications for executive functions. In the same vein, age of L2 acquisition could also explain discrepancies between the two studies. Early and late bilinguals might engage different cognitive and neural mechanisms during language processing, so that the effect of language context on executive control might not be comparable between the two types of bilinguals. Finally, it is worth noting that unlike Chinese and English, Welsh and English are both alphabetical languages. Switching between two languages with more similarities in linguistic structures might engage different executive components as compared to switching between two languages that differ more radically.

To verify that language contexts may exert different effects on the inhibitory control of bilinguals with different language backgrounds, the present study examined trilingual speakers while they performed a flanker task (Luk et al., 2010) following picture naming in different dual-language contexts: the L1-L2 context, the L2-L3 context, and the L1-L3 context. Within-subject comparisons of their performance in the flanker task following the three contexts will provide a more confident answer to the modulation effect of language context on inhibitory control. It is our hypothesis that in the L1-L2 context (Cantonese–Mandarin), as in Wu and Thierry (2013), bilingual context would facilitate inhibitory control performance; in the L2-L3 (Mandarin–English) and the L1-L3 (Cantonese–English) contexts, as in Liu et al. (2016), the bilingual context would have no beneficial effect on inhibitory control.

The second goal of the current study is to examine how dual-language contexts modulate the functional brain network for inhibitory control, which is usually right-lateralized. For this purpose, effective connectivity analyses, following a recently developed procedure for valid group modeling, namely Group Interactive Multiple Model Estimation (GIMME, Gates and Molenaar, 2012) was performed to identify causal relationships between key brain regions that subserve inhibitory control in different dual-language contexts. If dual-language contexts do not influence inhibitory control process, participants should display a typical flanker effect and comparable brain activation patterns as well as common functional brain network when performing the flanker task. If dual-language contexts do modulates inhibitory control, it is our hypothesis that the L1-L2 context would elicit a right-lateralized network for inhibitory control, while the L1-L3 and L2-L3 contexts might engage a less typical inhibitory control network, because of the more demanding task on linguistic processing and language control in the L2-L3 and L1-L3 contexts, relative to the L1-L2 context.

## Materials and Methods

### Participants

Thirty students (10 males; age range 18–25) were recruited from the Guangdong University of Foreign Studies in Guangzhou, a city with a large Cantonese–Mandarin bilingual community. All participants were highly proficient early bilinguals of Cantonese (first language, L1) and Mandarin (second language, L2): They were raised up in a Cantonese family and have acquired Mandarin since early childhood. At the time of testing, participants use both languages on a regular basis.

All participants were late learners of English (third language, L3) in the mainstream classroom and had a moderate level of proficiency. They started to learn English at an average age of 7.4 (±1.82). According to their self-report, English and Mandarin were used as the main instruction languages in their English class (English usage: 52% ± 0.22; Mandarin usage: 40% ± 0.2; Cantonese usage: 7% ± 0.11), implying considerable experiences of switching and translation between English and Mandarin as a result of English learning.

As shown in **Table 1**, to assess the participants’ linguistic knowledge and background variables in each of their three languages, we asked them to complete the following measures: (1) responses to the Language History Questionnaire (LHQ 2.0; Li et al., 2014) including the age of language acquisition (AoA), usage habits, switching frequency, and language abilities, (2) vocabulary knowledge in each language as examined through naming accuracy rates in a picture naming task (48 out of the 96 high-frequency non-living objects were selected as the stimuli from the battery of Snodgrass and Vanderwart (1980) and matched between languages, and (3) the Oxford Quick Placement Test (2001) as measurements of their English proficiency.

**Table 1:** Demographic variables, measures of cognitive skills, and language background information of the Cantonese–Mandarin–English trilingual participants.

	Cantonese–Mandarin–English Trilinguals (*n* = 30)
Age	21.64 ± 1.34
Handedness	42.77 ± 2.31
Non-verbal IQ (%)	74.17% ± 24.46
Working Memory (max:21)	20.03 ± 0.96
Processing Speed (ms)	1307.03 ± 180.21
**Language measures**	
Oxford Quick Placement Test (max:60)	45.4 ± 5.88
L1 picture naming (ACC)	99% ± 0.01
L2 picture naming (ACC)	100% ± 0.01
L3 picture naming (ACC)	95% ± 0.07
Language History Questionnaire (self-report)	
Reading in L1 (max:7)	6.7 ± 0.75
Writing in L1 (max:7)	6.17 ± 1.09
Speaking in L1 (max:7)	6.87 ± 0.43
Listening in L1 (max:7)	6.93 ± 0.25
Years of L1 learning	20.07 ± 1.41
Reading in L2 (max:7)	6.73 ± 0.58
Writing in L2 (max:7)	6.77 ± 0.57
Speaking in L2 (max:7)	6.57 ± 0.73
Listening in L2 (max:7)	6.87 ± 0.35
Years of L2 learning	17.6 ± 1.87
Reading in L3 (max:7)	5.7 ± 0.88
Writing in L3 (max:7)	5.13 ± 0.73
Speaking in L3 (max:7)	4.9 ± 0.96
Listening in L3 (max:7)	4.93 ± 0.83
Years of L3 learning	13.83 ± 2.15

*L1, the first language (Cantonese); L2, the second language (Mandarin); L3, the third language (English). RT: reaction time; ACC: accuracy rate.*

Based on language experience, usage habits, and language proficiency, the participants in the current study were characterized as (1) highly proficient in Cantonese and Mandarin, and with extensive experiences of switching between the two languages during conversations (i.e., in the L1-L2 context), and (2) moderately proficient in English but with more Mandarin–English switching experience (i.e., in the L2-L3 context) than Cantonese–English switching experience (i.e., in the L1-L3 context). All participants were right-handed as measured by the handedness inventory (Snyder and Harris, 1993). Written informed consent was obtained from all participants prior to the experiment. The Human Research Ethics Committee for Non-Clinical Faculties at the School of Psychology of South China Normal University approved this study. All subjects gave written informed consent in accordance with the Declaration of Helsinki.

### Materials and Procedure

#### Cognitive Assessments

Before the fMRI session, all participants received a battery of behavioral tests that are designed to measure their non-verbal intelligence (the Raven’s Standard Progressive Matrices; Raven et al., 1988) and working memory (the odd-even sequencing task, an adaptation of number-sequencing subtest form the WAIS-III; Wechsler, 1997) as shown in **Table 1**.

#### fMRI Procedure

**FIGURE 1:**
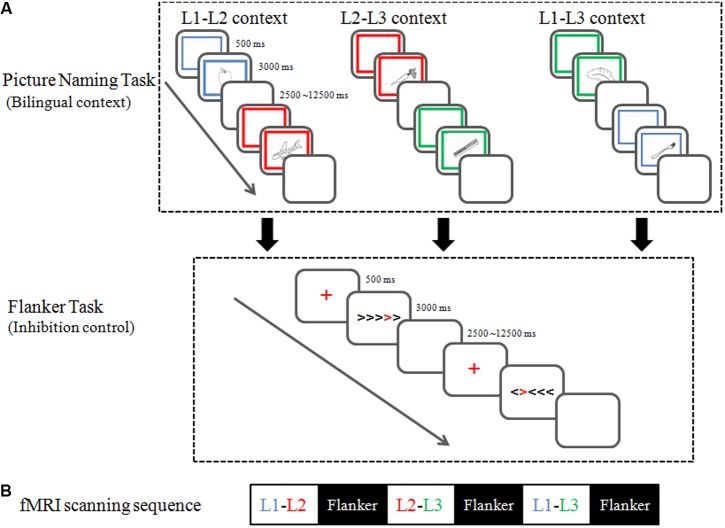
Experiment paradigm **(A)** and fMRI scanning sequence **(B)**.

Participants completed six event-related fMRI runs, each lasting 6 min and 36 s. As shown in **Figure 1B**, every picture-naming run was presented prior to a flanker run. The order of the three dual-language contexts (i.e., L1-L2, L2-L3, and L1-L3) was counterbalanced between participants in a Latin square design.

The Picture Naming Task: As shown in **Figure 1A**, picture-naming tasks in the three dual-language contexts followed the same paradigm, each involving two different languages. During the picture-naming task, participants named pictures in two languages alternatingly, with 24 pictures per language. The stimuli were randomly selected from 96 black and white drawings for concrete non-living objects (e.g., piano) in the UCSD International Picture Naming Project (IPNP) picture database^2^ (Bates et al., 2003). All stimuli corresponded to high frequency words in both Chinese (Liu et al., 2011) and English (Brysbaert and New, 2009), and were matched for word frequency (*t*_95_ = 0.4, *p* = 0.69) between the two languages. To ensure the familiarity of the object names, we asked an independent group of 35 individuals (age range: 18–21) from the same population to rate the familiarity of the object names on a 5-point Likert scale (1 = very infrequent; 5 = very frequent). There were no significant differences between the levels of familiarities of object names in the three languages [L1 = 4.0 ± 0.62, L2 = 4.13 ± 0.71, L3 = 3.94 ± 0.74; *F*_(2,54)_ = 2.65, *p*s > 0.05].

In each trial of the picture naming task, a frame was presented for 500 ms and then a picture of an object appeared in the center of the frame for 3000 ms, followed by a blank screen of 500 ms. The color of the frame (blue, red, and green) served as the naming cue (blue for Cantonese, red for Mandarin, and green for English). Participants were instructed to covertly name the picture in the target language within 3000 ms. The 48 picture-naming trials were presented in a pseudo-random order with a jittered inter-stimulus interval (min = 2000 ms, max = 12000 ms) optimized with OptSeq2 (Dale, 1999).^3^ During the inter-trial interval, a central fixation cross was presented. Due to equipment limitation and to minimize head movements during naming, naming responses were collected outside of the MRI scanner. As an orientation procedure, we informed participants that they would later complete a behavioral test related to the naming task inside the scanner. We collected behavioral data with the same task outside of the scanner 2 weeks after the fMRI sessions (e.g., Zou et al., 2012; Li et al., 2013).

The Flanker Task: Immediately following each picture-naming task, participants were scanned in a flanker task session, to examine the influence of language context on their inhibitory control process. During the flanker task (Luk et al., 2010), participants responded to the direction of a red chevron (i.e., the target) surrounded by other black chevrons (i.e., the flankers). As shown in **Figure 1A**, in congruent trials, flanker chevrons point in the same direction as the target, whereas in incongruent trials, flankers pointed in the opposite direction to the target. Twenty-four congruent trials and 24 incongruent trials were randomly presented during each flanker task scanning session, with jittered inter-stimulus intervals (min = 2000 ms, max = 12000 ms). Each trial began with the presentation of a red fixation for 500 ms, followed by the stimulus for 3000 ms, and then a blank buffer of 500 ms.

### MRI Acquisition

MRI images were acquired on a 3-T scanner (Siemens Trio Tim) equipped with a 12-channel phased-array head coil at the South China Normal University, using a T2^∗^-weighted gradient-echo EPI sequence (TE = 30 ms; TR = 2s; flip angle = 90°; slices = 32; matrix size = 64 × 64; FoV = 192 mm × 192 mm; thickness = 4 mm). Participants lay supine in the scanner and viewed the visual stimuli via a back-projection mirror, while their heads were immobilized with cushions. For each run, the functional scanning was always preceded by 6 s of dummy scans (fixation) to ensure tissue steady-state magnetization. High-resolution (1 mm × 1 mm × 1 mm) anatomical images were acquired using a T1-weighted, 3D inversion-recovery gradient-echo (MP-RAGE) sequence.

### Data Analyses

#### Whole Brain Activations

The fMRI data were preprocessed using the Statistical Parametric Mapping (SPM) software running under MATLAB (SPM12; Wellcome Department of Imaging Neuroscience, University College London).^4^ All three flanker runs followed the same data processing procedure. The first three scans (dummy scans) of the 198 volumes collected were discarded to allow for T1 equilibration. The remaining 195 volumes were then realigned to the first volume for head-motion correction, co-registered to the individual anatomical images and then to the EPI template in SPM12 based on the Montreal Neurological Institute (MNI) stereotactic space, and resampled into 3 mm × 3 mm × 3 mm cubic voxels. The head motion and rotation of all participants were less than 3 mm of displacement or 3° of rotation.

For each participant, functional images collected from each flanker run were grouped into congruent and incongruent conditions. Individual brain activations corresponding to congruent or incongruent conditions (in contrast to fixation) were analyzed using general linear model (GLM) and were entered into the second level of group analysis to show the neural correlates underlying inhibitory control.

AlphaSim program in the REST (Song et al., 2011) software was used to correct for multiple comparisons in SPM (10,000 interactions). All the brain activations reported below survived an FWE-corrected cluster-level threshold of *p* < 0.05 (single voxel: *p* < 0.001, number of voxels > 12) (Woo et al., 2014) and were in the MNI coordinate space.

#### ROI Selection and Analysis

Based on previous fMRI literature on language control and inhibitory control (Garavan et al., 1999; Luk et al., 2012; Grant et al., 2015; Abutalebi and Green, 2016), we selected 12 regions of interest (ROIs) to compose a cortical-subcortical-cerebellar network, which includes the right middle frontal gyrus (MFG) (33, 36, 21), right inferior frontal gyrus (IFG) (48, 9, 21), bilateral insula (INS) (±36, 3, -3), bilateral supramarginal gyri (SMG) (±18, 0, 21), bilateral caudate nuclei (CN) (±18, -21, 24), bilateral thalamus (THA) (±21, -30, 3), and bilateral cerebellum (CERE) (±15, -69, 42). Averaged time course data of all the voxels within a sphere (6 mm radius) in each ROI were extracted using the DPBABI software (Yan et al., 2016)^5^.

To identify activation changes in those regions between congruent and incongruent conditions following the three different dual-language contexts, the present study sorted time series of the 12 ROIs by experimental conditions (e.g., congruent and incongruent). The averaged time course signals across all trials of the congruent/incongruent conditions were converted to percentage signal changes (PSC) using the formula (signal-baseline)/baseline × 100 for each time point, and the baseline constant was the mean signal of the fixation baseline (e.g., Li et al., 2013). The averaged PSC values for each condition in every context were considered as representative activation level of each ROI for every participant.

#### Effective Connectivity

To examine the influence of dual-language context on functional brain connectivity of inhibitory control process in the trilinguals, we made use of recent advances in connectivity modeling (extended unified structural equation modeling, euSEM) (Gates et al., 2011; Hillary et al., 2011, 2014; Gates and Molenaar, 2012; Yang et al., 2015) and a recently developed Group Iterative Multiple Model Estimation (GIMME), an automatic and freely distributed MATLAB-based program^6^.

The euSEM approach has provided a flexible and efficient method for analyzing the causal interactions of brain regions for cognitive functions, as has previously been applied in Grant et al. (2015) and Yang et al. (2015). The procedure for using the euSEM in the current study is consistent with Yang et al. (2015), but with two experimental conditions in every language context, namely congruent and incongruent conditions of the flanker task. As with other SEM-based approaches, GIMME works from individual-level correlation matrices. The covariance matrices used for the euSEM analysis include the ROI time series at time t (contemporaneous series, where each “t” is a single brain volume or TR) and the same ROI time series at the next time t + 1 (lagged series). For the euSEM analysis, the covariance matrices also include two time series of the effects of the task inputs (congruent and incongruent) for both time t and t + 1, convolved with a canonical hemodynamic response function. In addition, the bilinear series can be used to measure the influence of task inputs on the relationship between ROIs by examining time series of each ROI at each time t multiplied by the convolved task input series at time t. The model selections at the group and individual levels are implemented in the following steps. First, Lagrange Multiplier equivalents (i.e., modification indices; Sörbom, 1989) are used to identify which effects (including connections among ROIs, the direct and bilinear effects), if freed, optimally improve model fit *across all individuals.* The probability of detecting an effect across all individuals was set at 75%; selection of this criterion was informed by empirical and simulated studies on the likelihood of detecting a true effect should it exist in a given sample (e.g., Hillary et al., 2011, 2014; Gates and Molenaar, 2012; Yang and Li, 2012; Yang et al., 2015). The program iterates until the 75% criterion is met. Second, the model is pruned by eliminating connections that are no longer significant for 75% of the group after other connections are freed. Third, individual-level models are estimated in a semi-confirmatory manner. All connections freed in the group model (described in the two steps above) are freed at the individual level. The automatic search procedure within LISREL (Cziráky, 2004) then iteratively frees connections that optimally improve model fit, according to the Lagrange Multiplier equivalents (Gates et al., 2010). Finally, the model is pruned by eliminating individual-level connections that become non-significant after other individual-level connections are freed, and a confirmatory model is fitted. Model fit parameters, that were found to demonstrate reliability in simulation studies (e.g., Gates et al., 2010) and fMRI studies (e.g., Hillary et al., 2014), were chosen *a priori* so that two of the following four criteria were satisfied in the final model: confirmatory fit index (CFI) ≥ 0.9; non-normed fit index (NNFI) ≥ 0.9.

## Results

### Behavioral Results

**FIGURE 2:**
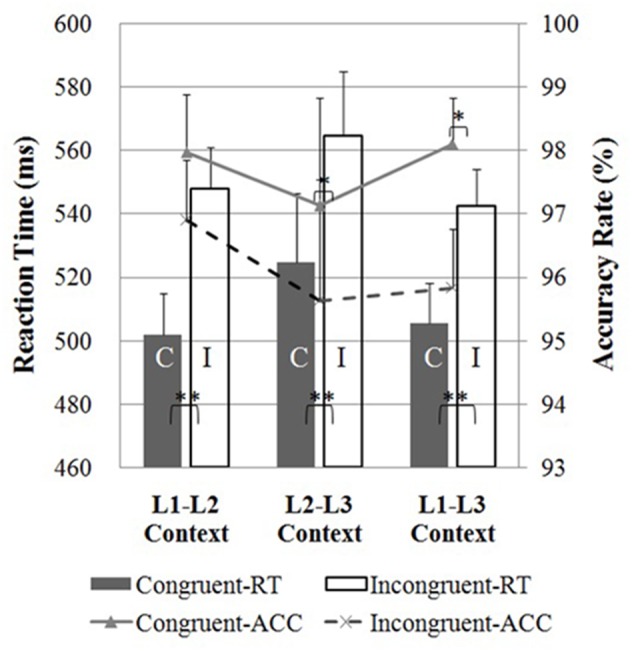
Reaction times (bars; left axis) and accuracy rates (lines; right axis) in the flanker task for the Cantonese-Mandarin-English trilinguals in the L1-L2, L2-L3, and L1-L3 contexts. Reaction times for the congruent condition (C) and incongruent condition (I) were significantly different in the three contexts. For accuracy rate, there was no significant difference between congruent and incongruent conditions when the flanker task was presented in the L1-L2 context. The asterisks indicate significant differences (^∗∗^*p* < 0.001; ^∗^*p* < 0.05). Error bars depict SEM in reaction time data.

As shown in **Figure 2**, participants performed more quickly in the congruent condition as compared to the incongruent condition, displaying a typical flanker interference effect (Eriksen and Eriksen, 1974) in all three dual-language contexts (*p*s < 0.001). However, there were no significant differences in response times between the congruent or incongruent trials following the three dual-language contexts (*p*s > 0.05).

For accuracy rates, the flanker interference effect (higher accuracy rates in the congruent as compared to the incongruent trials) was found following the L2-L3 context (two-sample *t*-test, *t*_29_ = 2.27, *p* < 0.05) and the L1-L3 context (*t*_29_ = 2.32, *p* < 0.05), but not the L1-L2 context (two-sample *t*-test, *t*_29_ = 1.14, *p* = 0.26), suggesting a facilitation effect of the L1-L2 context on inhibitory control process.

### fMRI Results

#### Whole-Brain Analysis

As shown in **Table 2**, in the L1-L2 context, incongruent trials elicited additional brain activations in the left inferior parietal lobe. In the L2-L3 context, incongruent conditions involved extra neural responses in the right inferior frontal gyrus and left supramarginal gyrus. In the L1-L3 context, both congruent and incongruent trials evoked neural activities in the right prefrontal cortex, right insula, and subcortical areas. See Supplementary Figure S1 for brain activations during the naming tasks.

**Table 2:** Whole brain activations associated with the flanker conditions for the Cantonese–Mandarin–English trilinguals in the L1-L2, L2-L3 and L1-L3 contexts.

	Congruent > fixation						Incongruent > fixation					
	Peak MNI Coordinate				Voxel		Peak MNI Coordinate				Voxel	
	BA	*x*	*y*	*z*	size	*t*	BA	*x*	*y*	*z*	size	*t*
**L1-L2 context**												
Insula	–	-42	0	9	32	5.1						
Postcentral gyrus	1	-63	-18	30	131	6.3	1	-54	-21	27	30	5.2
	1	60	-15	24			1	63	-15	21	18	5.3
Supramarginal gyrus							40	-60	-21	42	12	4.9
Fusiform gyrus	37	27	-48	-18	1857	8.9						
Lingual gyrus							18	15	-87	-12	1437	9.1
Thalamus	-	-21	-30	3	15	7.1	-	-21	-30	3	15	6.7
**L2-L3 context**												
Inferior frontal gyrus							44	48	9	21	15	5.5
Insula	–	-36	3	-3	16	4.7						
	–	36	6	3	15	5.0	–	45	9	-3	53	5.9
Rolandic operculum							–	-39	-3	15	25	5.5
Postcentral gyrus	1	-60	-18	24	52	5.1						
Supramarginal gyrus	40	57	-21	21	42	4.8	40	-63	-21	21	52	5.8
							40	57	-21	21	180	5.6
Lingual gyrus							18	15	-78	-12	2061	7.7
Fusiform gyrus	37	21	-48	-15	2037	9.6						
Thalamus	–	24	-30	0	15	6.7	-	24	-30	0	19	6.5
**L1-L3 context**												
Middle frontal gyrus	9	33	36	21	17	5.0	9	33	36	21	23	5.3
Inferior frontal gyrus	44	60	15	15	29	4.9	44	51	15	0	12	4.3
							45	45	39	3	26	5.2
Insula	–	-27	-3	-9	13	4.9						
	–	39	3	0	14	5.5	–	36	0	12	18	4.6
Rolandic operculum							–	-48	-9	12	29	5.4
Postcentral gyrus	1	-54	-21	39	119	5.7						
	1	54	-24	54	21	5.2						
Heschl gyrus	13	-48	-12	9	33	5.1						
Superior parietal lobule							7	21	-69	57	24	5.2
Inferior parietal lobule							40	-48	-39	57	234	6.2
Precuneus							7	12	-48	75	16	4.2
Supramarginal gyrus							40	60	-18	27	212	7.0
							40	30	-39	42	21	4.4
Lingual gyrus	18	9	-78	-9	1722	8.1	18	9	-78	-9	2371	8.4
Caudate	–	-18	-15	24	25	5.6	–	-18	-21	24	22	5.0
	–	18	-21	21	25	5.6	–	18	-15	24	14	5.1
Putamen	–	27	-3	-12	18	5.3		-30	-12	-9	24	5.3
							–	33	-15	-6	16	6.3
Hippocampus							–	-24	-30	-3	15	5.3
							–	18	-30	-3	32	6.4
Cerebellum	–	15	-72	-42	12	4.7	–	-3	-69	-36	14	4.9
							–	15	-69	-42	14	4.4

**FIGURE 3:**
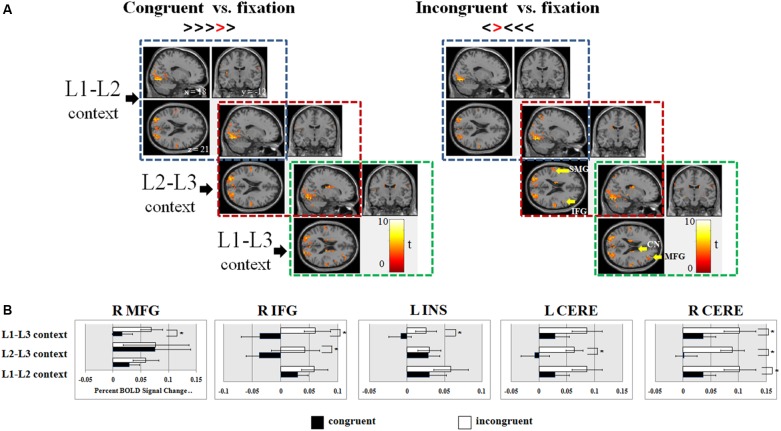
**(A)** Overall brain activations associated with congruent and incongruent trials of the flanker task as presented in the L1-L2, L2-L3, and L1-L3 contexts; **(B)** Regions of interests that were sensitive to the flanker effect in different dual-language contexts. MFG, middle frontal gyrus; IFG, inferior frontal gyrus; INS, insula; CN, caudate nucleus; THA, thalamus; SMG, supramarginal gyrus; CERE, cerebellum; L, left hemisphere; R, right hemisphere; ^∗^*p* < 0.05.

No significant differences of neural activations were found between congruent and incongruent conditions in the L1-L2 and L2-L3 contexts. In the L1-L3 context, incongruent condition, as compared to the congruent condition, showed more neural responses in the bilateral inferior occipital gyri (Brodmann area 19, or BA 19), right middle occipital gyrus and bilateral middle temporal gyri (MTG). It is well-known that the medial temporal lobe (MTL) is the hub for declarative memory and keeps semantic representation (Squire et al., 2004). MTG might be a multimodal semantic processing hub, storing long-term conceptual knowledge, processing lexico-semantic information, and fulfilling semantic integration, especially in the L2 lexical processing (Rodríguez-Fornells et al., 2009). The stronger activation of the bilateral MTG in incongruent trials following L1-L3 context might imply competition of cognitive resources between inhibitory control task and the demanding semantic processing in L1-L3 context. As illustrated in **Figure 3A**, dual-language contexts (i.e., the L2-L3 and L1-L3 contexts) involving a moderate proficient language (L3) displayed increased brain activity in the right prefrontal cortex, bilateral insula and inferior parietal lobules, as well as subcortical areas, particularly the bilateral caudate and putamen, as compared to the L1-L2 context.

#### ROI Analyses

The following 12 ROIs were chosen based on the extant imaging literature of inhibitory control and language control (see ROI Selection in the Section of Materials and Methods): The right MFG, right IFG, bilateral INS, SMG, CN, Thalamus (THA), as well as bilateral cerebellum (CERE). Our analyses of the percent BOLD signal changes in those ROIs found (1) significant flanker effects in all the three dual-language contexts in the right cerebellum [right CERE, *F*_(1,29)_ = 15.56, *p* < 0.001]; (2) brain activations in the right IFG and left cerebellum were associated with the flanker effect in the L2-L3 context [right IFG, *F*_(1,29)_ = 5.65, *p* = 0.024; left CERE, *F*_(1,29)_ = 6.19, *p* = 0.019]; (3) neural responses in the right cerebellum, right MFG, right IFG, and left INS were associated with the flanker effect only in the L1-L3 context [MFG, *F*_(1,29)_ = 7.09, *p* = 0.013; IFG, *F*_(1,29)_ = 58.75, *p* = 0.006; INS, *F*_(1,29)_ = 4.37, *p* = 0.045] (**Figure 3B**).

#### Connectivity Analysis

**FIGURE 4:**
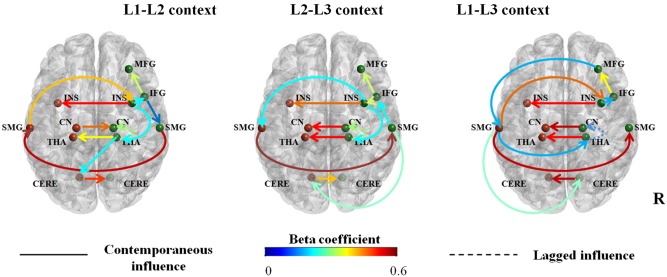
Connectivity maps for inhibitory control process in three dual-language contexts (L1-L2; L2-L3; L1-L3). Significant contemporaneous and lagged relationships at group-level were shown, and auto-regressive lagged connections are omitted for clarity. Connection strength is denoted by beta coefficients, reflected here as the color the line. The solid line indicates contemporaneous relationship, namely area X at time T influences brain activation of area Y at time T; dotted line illustrates lagged relationship, namely area X at time T influences brain activation of area Y at time T + 1. Nodes (ROIs) are MFG, middle frontal gyrus; IFG, inferior frontal gyrus; INS, insula; CN, caudate nucleus; THA, thalamus; SMG, supramarginal gyrus; CERE, cerebellum; R, right hemisphere.

An extended unified Structural Equation Model (euSEM) analysis was conducted on the fMRI data of the flanker tasks following the L1-L2, L2-L3 and L1-L3 contexts. All group maps (**Figure 4**) had an excellent fit to the data for roughly 97–100% of the participants, depending on the measure (Brown, 2006). Specifically, in the L1-L2 context, the Comparative Fit Index (CFI) evaluated the model fit as excellent for 100% of the participants’ data, while Standardized ROOT Mean Square Residual (SRMR) and the Root Mean Square Error of Approximation (RMSEA) showed excellent fit for 97% of the data. In L2-L3 context, CFI, SRMR and the RMSEA results indicated an excellent fit for 100% of the data, as is the same in the L1-L3 context.

As shown in **Figure 4**, the cortical-subcortical-cerebellar network for inhibitory control following the three dual-language contexts shared common connections: The right INS and thalamus strongly influence their left homologous areas, implying the right-dominant network for inhibitory control; the right IFG feeds to the right MFG, suggesting that the right IFG is highly engaged in bottom–up process of inhibitory control; the right thalamus influences brain activations in the right caudate in a lagged relationship, implying the key role of right thalamus in the communications between cortical and subcortical areas. In all the three contexts, right INS influences right IFG and left SMG. Not surprisingly, the left and right SMG are connected to each other as for the bilateral caudate nuclei.

Obviously, in all three dual-language contexts, inhibitory control relied on collaborations between a frontal-parietal network, a cortical-thalamic-striato pathway and bilateral cerebellum. However, dual-language contexts influence inhibitory control network in the trilinguals. To be specific, inhibitory control in the L1-L2 context recruits an efficient and right lateralized network: The right INS as the hub of the network feeds to the right IFG, modulating brain activation in the right MFG indirectly and activating the right SMG; the right THA, as a mediator of frontal-thalamic-striato pathway, receiving positive information from the right INS, feeds to the right CN; right THA is also a key relay station for cortico-cerebellar pathway, forwarding information from the right INS to the left cerebellum, which sends strong and positive influence to the right cerebellum; right SMG forward information from the right IFG to the left SMG, which passes information to the right insula, completing the frontal-parietal sub-network.

When the dual-language context involves a moderate proficient language (L3), the functional brain network for inhibitory control in the same group of participants changed immediately. Specifically, in the L2-L3 context, the frontal-parietal sub-network runs in the reverse pipeline: The right IFG actively influences the right INS, which as a hub sends direct and positive information to the left SMG, feeding directly to the right SMG; the right IFG receives feedback from the right SMG and connects to the right MFG. As in the L1-L2 context, right INS influences bilateral caudate via THA in a lagged relationship. The left cerebellum receives inputs directly from the right SMG and feeds to the right cerebellum. Interestingly, the right CN activates left CN in the L2-L3 context, as is the same in the condition of the L1-L3 context.

L1-L3 context involves a moderate proficient language and engages less switching experience between the two languages. Compared with the L1-L2 and L2-L3 contexts, inhibition control in the L1-L3 context relies on a more left-lateralized and less integrated network, compared with the networks in the other two dual-language contexts. In L1-L3 context, the left SMG works as the hub of the inhibitory control network: The right MFG takes the lead and sends orders directly to the left SMG, which relays to the right INS, right SMG, right THA, and right cerebellum. The right THA, as in the other two conditions, forward positive information to the right CN in a lagged relationship, thus completing the cortico-thalamic-striato pathway. For the cerebellar components, the right cerebellum receives weak influence from SMG on the contralateral side and feeds to the left cerebellum.

Taken together, the cortical-subcortical-cerebellar network for inhibitory control involves a frontal-parietal sub-network, cortico-thalamic-striato pathway, and bilateral cerebellum. However, this neural pattern can be modulated by language contexts on a short timescale. In dual-language contexts with intensive code switching between two proficient languages (i.e., the L1-L2 context), the inhibitory control process seems to be facilitated and rely on a right-lateralized control network. When the dual-language context involves a less proficient language, especially when the two languages are radically different from each other, the inhibitory control process relies on a more left-lateralized and less integrated neural network: In the L2-L3 context, the right IFG feeds to right INS and receives feedback from the right SMG; in the L1-L3 context, the right MFG is highly engaged and the whole network relies on the left SMG to modulate brain activations in the cortical, subcortical, and cerebellar areas.

## Discussion

The current study examines the dynamic influences of language contexts on inhibitory control in trilinguals. We explored the neural correlates and functional brain networks activated while Cantonese–Mandarin–English trilingual speakers performed the flanker tasks in three dual-language contexts. As bilingual language processing engages inhibition of the non-target language (Dijkstra and Van Heuven, 1998; Green, 1998; Linck et al., 2008; Green and Abutalebi, 2013; Ventura-Campos et al., 2013; Abutalebi and Green, 2016) and acquiring a second language facilitates the development of cognitive control (Linck et al., 2009; Hosoda et al., 2013; Grant et al., 2015), we expected a facilitatory effect of dual-language contexts on participants’ cognitive control performance (e.g., Wu and Thierry, 2013). Our participants were highly proficient in L1 and L2, but moderately proficient in L3. They frequently switch between L1 and L2 in everyday life, but not between L1 and L3 or L2 and L3. We, therefore, expected to observe significant facilitatory effect of the L1-L2 context as compared to the L2-L3 and L1-L3 contexts on inhibitory control.

The results showed the classic flanker effect in the L2-L3 and L1-L3 contexts, but not in the L1-L2 context. Consistent with results in a previous study (Wu and Thierry, 2013), the effect of contextual priming was observed in accuracy rates but not reaction times, suggesting that independent cognitive mechanisms might account for flanker effects in the two types of measurements. Previous studies (e.g., Luk et al., 2010) using a similar flanker task showed that in the incongruent condition, bilinguals activated a widespread set of brain regions, including the fusiform gyri, inferior frontal gyri, supplementary motor area, inferior parietal regions, and subcortical areas. In the present study, those brain regions for inhibitory control failed to show significant activations in incongruent condition (when compared to the congruent condition) in the L1-L2 context. Moreover, none of the ROIs showed significant interference effects in the L1-L2 context in terms of their BOLD signal change (**Figure 3B**). These findings suggest that the neural efficiency of the inhibitory control network was enhanced in the L1-L2 context, reducing the classic flanker effect in both the behavioral and the neural anatomical level (e.g., Jäncke et al., 2000; Stevens et al., 2007). Effective connectivity analysis shows the following pattern of results: The right insula functions as the hub of the frontal-parietal network, feeding to the right IFG, which mediates the right MFG and bilateral SMG; the right MFG and bilateral SMG then send information to the right THA, which positively modulates brain activations of bilateral caudate nuclei in a lagged relationship and directly influences the cerebellar pathway. The important role of the right insula in inhibitory control has been well documented. In an event-related fMRI study, Garavan et al. (1999) showed that performing a response inhibition task activated the right hemisphere, including the right MFG, IFG, insula, and inferior parietal lobule. A more recent study dissociated the functional role of the right IFG and insula in inhibitory control and suggests that the right insula is particularly important for detecting behaviorally salient events, while the right IFG is more involved in implementing inhibitory control (Cai et al., 2014). Meanwhile, it is interesting to note that the right frontal-insular cortex has been implicated in switching between central-executive and default-mode networks (Sridharan et al., 2008).

In the L2-L3 context, behavioral results showed significant flanker effects in both reaction times and accuracy rates (see **Figure 2**). Analysis of the neuroimaging data showed that the flanker effect was associated with brain activations in the right inferior frontal gyrus, bilateral insula, left rolandic operculum, bilateral supramarginal gyrus, and right thalamus. This pattern of activations is highly consistent with neural mechanisms underlying typical flanker effects (e.g., Luk et al., 2010). ROI analyses showed that the flanker effect was associated with activations in the right IFG and left cerebellum, where no such significant activations were found in the L1-L2 context, suggesting a priming effect on the inhibitory control when the dual-language context involves two highly proficient languages. Connectivity map shows a similar frontal-parietal network, but in the reverse relationship. To be specific, in the L1-L2 context, the right IFG sends feedback to the right MFG and completes the frontal-parietal circuitry via the right SMG and left SMG; in the L2-L3 context, flanker task involves more engagement of the IFG: The IFG passes positive influences to the right INS, forwarding information to the left SMG and the right THA; the right SMG receives signals from the left SMG and sends feedback to the IFG, completing the frontal-parietal loop.

In the L1-L3 context, the flanker effect is associated with a different neural network, in which the left SMG is the hub. As illustrated in **Figure 4**, the right MFG influences the left SMG, which influences the right INS, the right IFG, and finally the right MFG, completing a frontal-insula-parietal network without the right SMG; meanwhile, the left SMG directly modulates brain activations in the right THA, which communicates with the right CN in a lagged relationship as in the other two contexts; furthermore, the left SMG feeds to the right cerebellum, which connects to the left cerebellum. This distinction of the neural network in the L1-L3 context, as compared to the L1-L2 and the L2-L3 contexts, is further supplemented by increased activations in the right MFG, right IFG, bilateral basal ganglia and cerebellum (see **Table 2**). ROI analyses also showed that activations in the right MFG and right cerebellum were associated with the flanker effect.

Research in neuropsychology and cognitive neuroscience has established the role of the left supramarginal gyrus in the inferior parietal lobule in second language acquisition. Neuroimaging data showed that early bilingualism is associated with increased gray matter density in the left inferior parietal lobe (Mechelli et al., 2004). In addition, researchers have found that the lateral inferior parietal cortex contributes to attentional focalization and target detection in both auditory and visual modalities, indicating its involvement in domain-general attentional processes (e.g., Green et al., 2006; Shomstein and Yantis, 2006). As summarized by Della Rosa et al. (2013), second language acquisition might tune this attentional control area into a “multilingual talent area” as phonological storage and attentional control functions were also subserved by this left inferior parietal lobe. The right SMG, as suggested by Abutalebi and Green (2008), was particularly involved in language selection in conversations that involve multiple languages. As illustrated in **Figure 4**, in the L1-L2 context, the right SMG is influenced by the right IFG in a top-down control process, while in the L2-L3 context, it sends feedback to the right IFG, forming a bottom–up stream.

It is worth noticing that in the L1-L2 context, consistent with the adaptive control hypothesis (Abutalebi and Green, 2016), the right IFG feeds to the right insula, which influences the right thalamus, thus modulating subcortical areas such as the caudate and connecting the cerebellum. The thalamus has been reported to directly connect to the regions of basal ganglia (Smith et al., 2011) and has reciprocal structure connections with the cerebellum as a relay station (e.g., Glickstein and Doron, 2008). The left caudate and putamen might be more involved in verb interference effects (Abutalebi and Green, 2008; Ali et al., 2010), while the right homologous areas play a more important role in inhibitory control. Based on our results of trilinguals, dual-language contexts modulate the involvement of inhibitory control areas and their interactions.

## Conclusion

The finding that dual-language contexts lead to functional reorganizations of the inhibitory control network not only reconciles discrepancies in previous studies (e.g., Wu and Thierry, 2013; Liu et al., 2016), but also provides a novel perspective for investigating the interplay between language control and non-linguistic cognitive processes. To fully understand the nature of the neural mechanisms subserving non-linguistic skills (e.g., executive functions), researchers have to consider the influences of processing contexts. Results of the current study provides empirical evidence in favor of the adaptive control hypothesis (Green and Abutalebi, 2013), which suggests that interactional contexts (e.g., single-language, dual-language, or frequent-switching) modulate language control processes by adaptive changes in the neural regions and circuits associated with specific control processes. Critically, our results showed that the cognitive system and its underlying neural network are highly plastic, allowing quick development of functional reconfigurations. Short-term language engagement, in the form of contextual priming, can instantly rewire the related brain mechanisms. This finding sheds new light on therapy training programs for individuals with minor cognitive impairment (MCI). Whether or not L2 proficiency, age of acquisition, and cross-language similarities (e.g., alphabetical or non-alphabetical) distinctly contribute to the modulation effects of bilingual contexts requires further exploration.

## Author Contributions

JYa, JYe, RW, KZ, and YW designed the study. JYa and JYe acquired and analyzed the data. JYa, RW, KZ, and YW wrote the manuscript.

## Conflict of Interest Statement

The authors declare that the research was conducted in the absence of any commercial or financial relationships that could be construed as a potential conflict of interest.

## Footnotes

1 Although Mandarin and Cantonese are often referred to as two dialects of Chinese, they are mutually unintelligible in their oral forms (Tang and van Heuven, 2009), and significantly different in phonology, lexicon, and syntax (Cai et al., 2011). Therefore, in the literature of bilingualism, Mandarin and Cantonese represent two distinct languages, rather than two dialects (e.g., Cai et al., 2011; Tu et al., 2015; Liu et al., 2017).

2 http://crl.ucsd.edu/aszekely/ipnp/

3 http://surfer.nmr.mgh.harvard.edu/optseq/

4 http://www.fil.ion.ucl.ac.uk/spm

5 http://rfmri.org/dpabi

6 www.mathworks.com

## References

[B1] Abutalebi J. Della Rosa P. A. Ding G. Weekes B. Costa A. Green D. W. (2013a). Language proficiency modulates the engagement of cognitive control areas in multilinguals. *Cortex* 49 905–911. 10.1016/j.cortex.2012.08.018 23021069

[B2] Abutalebi J. Della Rosa P. A. Gonzaga A. K. Keim R. Costa A. Perani D. (2013b). The role of the left putamen in multilingual language production. *Brain Lang.* 125 307–315. 10.1016/j.bandl.2012.03.009 22538086

[B3] Abutalebi J. Green D. W. (2007). Bilingual language production: the neurocognition of language representation and control. *J. Neurolinguistics* 20 242–275. 10.1016/j.jneuroling.2006.10.003

[B4] Abutalebi J. Green D. W. (2008). Control mechanisms in bilingual language production: neural evidence from language switching studies. *Lang. Cogn. Process.* 23 557–582. 10.1080/01690960801920602 24795491

[B5] Abutalebi J. Green D. W. (2016). Neuroimaging of language control in bilinguals: neural adaptation and reserve. *Bilingualism* 19 689–698. 10.1017/S1366728916000225

[B6] Ali N. Green D. W. Kherif F. Devlin J. T. Price C. J. (2010). The role of the left head of caudate in suppressing irrelevant words. *J. Cogn. Neurosci.* 22 2369–2386. 10.1162/jocn.2009.21352 19803688 PMC3646394

[B7] Bates E. D’Amico S. Jacobsen T. Székely A. Andonova E. Devescovi A. (2003). Timed picture naming in seven languages. *Psychon. Bull. Rev.* 10 344–380. 10.3758/BF03196494 12921412 PMC3392189

[B8] Bercury K. K. Macklin W. B. (2015). Dynamics and mechanisms of CNS myelination. *Dev. Cell* 32 447–458. 10.1016/j.devcel.2015.01.016 25710531 PMC6715306

[B9] Bialystok E. (2007). Acquisition of literacy in bilingual children: a framework for research. *Lang. Learn.* 57 45–77. 10.1111/j.1467-9922.2007.00412.x 25669019

[B10] Bialystok E. Craik F. Luk G. (2008). Cognitive control and lexical access in younger and older bilinguals. *J. Exp. Psychol. Learn. Mem. Cogn.* 34 859–873. 10.1037/0278-7393.34.4.859 18605874

[B11] Bialystok E. Craik F. I. Grady C. Chau W. Ishii R. Gunji A. (2005). Effect of bilingualism on cognitive control in the Simon task: evidence from MEG. *Neuroimage* 24 40–49. 10.1016/j.neuroimage.2004.09.044 15588595

[B12] Bialystok E. Craik F. I. Klein R. Viswanathan M. (2004). Bilingualism, aging, and cognitive control: evidence from the Simon task. *Psychol. Aging* 19 290–303. 10.1037/0882-7974.19.2.290 15222822

[B13] Bialystok E. Craik F. I. M. Ryan J. (2006). Executive control in a modified antisaccade task: effects of aging and bilingualism. *J. Exp. Psychol.* 32 1341–1354. 10.1037/0278-7393.32.6.1341 17087588

[B14] Brown T. A. (2006). *Confirmatory Factor Analysis for Applied Research.* New York, NY: The Guilford Press.

[B15] Brysbaert M. New B. (2009). Moving beyond Kuèera and Francis: a critical evaluation of current word frequency norms and the introduction of a new and improved word frequency measure for American English. *Behav. Res. Methods* 41 977–990. 10.3758/BRM.41.4.977 19897807

[B16] Cai W. Ryali S. Chen T. Li C.-S. R. Menon V. (2014). Dissociable roles of right inferior frontal cortex and anterior Insula in inhibitory control: evidence from intrinsic and task-related functional parcellation, connectivity, and response profile analyses across multiple datasets. *J. Neurosci.* 34 14652–14667. 10.1523/JNEUROSCI.3048-14.2014 25355218 PMC4212065

[B17] Cai Z. G. Pickering M. J. Yan H. Branigan H. P. (2011). Lexical and syntactic representations in closely related languages: evidence from Cantonese-mandarin bilinguals. *J. Mem. Lang.* 65 431–445. 10.1016/j.jml.2011.05.003

[B18] Costa A. Hernández M. Sebastián-Gallés N. (2008). Bilingualism aids conflict resolution: evidence from the ANT task. *Cognition* 106 59–86. 10.1016/j.cognition.2006.12.013 17275801

[B19] Cziráky D. (2004). LISREL 8.54: a program for structural equation modelling with latent variables. *J. Appl. Econ.* 19 135–141. 10.1002/jae.767

[B20] Dale A. M. (1999). Optimal experimental design for event-related fMRI. *Hum. Brain Mapp.* 8 109–114. 10.1002/(SICI)1097-0193(1999)8:2/3<109::AID-HBM7>3.0.CO;2-W 10524601 PMC6873302

[B21] De Groot A. M. B. (2012). “Bilingualism and cognition,” in *The Encyclopedia of Applied Linguistics*, ed. Chapelle C. A. (Hoboken, NY: Blackwell Publishing Ltd.).

[B22] Delekate A. Zagrebelsky M. Kramer S. Schwab M. E. Korte M. (2011). NogoA restricts synaptic plasticity in the adult hippocampus on a fast time scale. *Proc. Natl. Acad. Sci. U.S.A.* 108 2569–2574. 10.1073/pnas.1013322108 21262805 PMC3038770

[B23] Della Rosa P. A. Videsott G. Borsa V. M. Canini M. Weekes B. S. Franceschini R. (2013). A neural interactive location for multilingual talent. *Cortex* 49 605–608. 10.1016/j.cortex.2012.12.001 23294573

[B24] Dijkstra T. Van Heuven W. J. B. (1998). “The BIA model and bilingual word recognition,” in *Localist Connectionist Approaches to Human Cognition*, eds Grainger J. Jacobs A. M. (London: Psychology Press), 189–225.

[B25] Dong Y. Zhong F. (2017). Interpreting experience enhances early attentional processing, conflict monitoring and interference suppression along the time course of processing. *Neuropsychologia* 95 193–203. 10.1016/j.neuropsychologia.2016.12.007 27939366

[B26] Eriksen B. A. Eriksen C. W. (1974). Effects of noise letters upon the identification of a target letter in a nonsearch task. *Percept. Psychophys.* 16 143–149. 10.3758/BF03203267

[B27] Fields R. D. (2005). Myelination: an overlooked mechanism of synaptic plasticity? *Neuroscientist?* 11 528–531. 16282593 10.1177/1073858405282304 PMC1474837

[B28] Garavan H. Ross T. J. Stein E. A. (1999). Right hemispheric dominance of inhibitory control: an event-related functional MRI study. *Proc. Natl. Acad. Sci. U.S.A.* 96 8301–8306. 10.1073/pnas.96.14.8301 10393989 PMC22229

[B29] Gates K. M. Molenaar P. Hillary F. G. Ram N. Rovine M. J. (2010). Automatic search for fMRI connectivity mapping: an alternative to Granger causality testing using formal equivalences among SEM path modeling, VAR, and unified SEM. *Neuroimage* 50 1118–1125. 10.1016/j.neuroimage.2009.12.117 20060050

[B30] Gates K. M. Molenaar P. C. Hillary F. G. Slobounov S. (2011). Extended unified SEM approach for modeling event-related fMRI data. *Neuroimage* 54 1151–1158. 10.1016/j.neuroimage.2010.08.051 20804852

[B31] Gates K. M. Molenaar P. C. M. (2012). Group search algorithm recovers effective connectivity maps for individuals in homogeneous and heterogeneous samples. *Neuroimage* 63 310–319. 10.1016/j.neuroimage.2012.06.026 22732562

[B32] Ghazi Saidi L. Perlbarg V. Marrelec G. Pélégrini-Issac M. Benali H. Ansaldo A. I. (2013). Functional connectivity changes in second language vocabulary learning. *Brain Lang.* 124 56–65. 10.1016/j.bandl.2012.11.008 23274799

[B33] Glickstein M. Doron K. (2008). Cerebellum: connections and functions. *Cerebellum* 7 589–594. 10.1007/s12311-008-0074-4 19002543

[B34] Grant A. M. Fang S.-Y. Li P. (2015). Second language lexical development and cognitive control: a longitudinal fMRI study. *Brain Lang.* 144 35–47. 10.1016/j.bandl.2015.03.010 25899988

[B35] Green D. W. (1998). Mental control of the bilingual lexico-semantic system. *Biling. Lang. Cogn.* 1 67–81. 10.1017/S1366728998000133 29368141

[B36] Green D. W. (2011). Language control in different contexts: the behavioral ecology of bilingual speakers. *Front. Psychol.* 2:103. 10.3389/fpsyg.2011.00103 21779260 PMC3132677

[B37] Green D. W. Abutalebi J. (2013). Language control in bilinguals: the adaptive control hypothesis. *J. Cogn. Psychol.* 25 515–530. 10.1080/20445911.2013.796377 25077013 PMC4095950

[B38] Green D. W. Crinion J. Price C. J. (2006). Convergence. Degeneracy, and control. *Lang. Learn.* 56 99–125. 10.1111/j.1467-9922.2006.00357.x 18273402 PMC2241761

[B39] Hillary F. G. Medaglia J. D. Gates K. Molenaar P. C. Slocomb J. Peechatka A. (2011). Examining working memory task acquisition in a disrupted neural network. *Brain* 134(Pt 5), 1555–1570. 10.1093/brain/awr043 21571783

[B40] Hillary F. G. Medaglia J. D. Gates K. M. Molenaar P. C. Good D. C. (2014). Examining network dynamics after traumatic brain injury using the extended unified SEM approach. *Brain Imaging Behav.* 8 435–445. 10.1007/s11682-012-9205-0 23138853

[B41] Hosoda C. Tanaka K. Nariai T. Honda M. Hanakawa T. (2013). Dynamic neural network reorganization associated with second language vocabulary acquisition: a multimodal imaging study. *J. Neurosci.* 33 13663–13672. 10.1523/JNEUROSCI.0410-13.2013 23966688 PMC6618649

[B42] Jäncke L. Shah N. Jand Peters M. (2000). Cortical activations in primary and secondary motor areas for complex bimanual movements in professional pianists. *Cogn. Brain Res.* 10 177–183. 10.1016/S0926-6410(00)00028-8 10978706

[B43] Kroll J. F. Bialystok E. (2013). Understanding the consequences of bilingualism for language processing and cognition. *J. Cogn. Psychol.* 25 497–514. 10.1080/20445911.2013.799170 24223260 PMC3820916

[B44] Li P. Zhang F. Tsai E. Puls B. (2014). Language history questionnaire (LHQ 2.0): a new dynamic web-based research tool. *Biling. Lang. Cogn.* 17 673–680. 10.1017/S1366728913000606

[B45] Li Y. Yang J. Suzanne Scherf K. Li P. (2013). Two faces, two languages: an fMRI study of bilingual picture naming. *Brain Lang.* 127 452–462. 10.1016/j.bandl.2013.09.005 24129199

[B46] Linck J. A. Hoshino N. Kroll J. F. (2008). Cross-language lexical processes and inhibitory control. *Ment. Lex.* 3 349–374. 10.1075/ml.3.3.06lin 19907674 PMC2774929

[B47] Linck J. A. Kroll J. F. Sunderman G. (2009). Losing access to the native language while immersed in a second language: evidence for the role of inhibition in second-language learning. *Psychol. Sci.* 20 1507–1515. 10.1111/j.1467-9280.2009.02480.x 19906121 PMC2858781

[B48] Liu C. Lu J. Sun X. Wang R. (2016). Immediate effect of language switch on non-proficient bilinguals’ cognitive control components. *Acta Psychol. Sin.* 48 472–481. 10.3724/SP.J.1041.2016.00472

[B49] Liu X. Tu L. Wang J. Jiang B. Gao W. Pan X. (2017). Onset age of L2 acquisition influences language network in early and late Cantonese-Mandarin bilinguals. *Brain Lang.* 174 16–28. 10.1016/j.bandl.2017.07.003 28711720

[B50] Liu Y. Hao M. Li P. Shu H. (2011). Timed picture naming norms for Mandarin Chinese. *PLoS One* 6:e16505. 10.1371/journal.pone.0016505 21298065 PMC3027682

[B51] Luk G. Anderson J. A. Craik F. I. Grady C. Bialystok E. (2010). Distinct neural correlates for two types of inhibition in bilinguals: response inhibition versus interference suppression. *Brain Cogn.* 74 347–357. 10.1016/j.bandc.2010.09.004 20965635

[B52] Luk G. Green D. W. Abutalebi J. Grady C. (2012). Cognitive control for language switching in bilinguals: a quantitative meta-analysis of functional neuroimaging studies. *Lang. Cogn. Process.* 27 1479–1488. 10.1080/01690965.2011.613209 24795491 PMC4006828

[B53] Martin-Rhee M. M. Bialystok E. (2008). The development of two types of inhibitory control in monolingual and bilingual children. *Biling. Lang. Cogn.* 11 81–93. 10.1017/S1366728907003227 25935936

[B54] Mechelli A. Crinion J. T. Noppeney U. O’Doherty J. Ashburner J. Frackowiak R. S. (2004). Neurolinguistics: structural plasticity in the bilingual brain. *Nature* 431 757–757. 10.1038/431757a 15483594

[B55] Morton J. B. Harper S. N. (2007). What did Simon say? Revisiting the bilingual advantage. *Dev. Sci.* 10 719–726. 10.1111/j.1467-7687.2007.00623.x 17973787

[B56] Oxford Quick Placement Test (2001). *Oxford Quick Placement Test.* Oxford: Oxford University Press.

[B57] Raven J. C. Raven J. Court J. H. (1988). *The Mill Hill Vocabulary Scale.* London: H. K. Lewis.

[B58] Rodríguez-Fornells A. Cunillera T. Mestres-Missé A. de Diego-Balaguer R. (2009). Neurophysiological mechanisms involved in language learning in adults. *Philos. Trans. R. Soc. Lond. B Biol. Sci.* 364 3711–3735. 10.1098/rstb.2009.0130 19933142 PMC2846313

[B59] Sabbagh C. Resh N. Mor M. Vanhuysse P. (2006). Spheres of justice within schools: reflections and evidence on the distribution of educational goods. *Soc. Psychol. Educ.* 9 97–118. 10.1007/s11218-005-3319-9

[B60] Shomstein S. Yantis S. (2006). Parietal cortex mediates voluntary control of spatial and nonspatial auditory attention. *J. Neurosci.* 26 435–439. 10.1523/JNEUROSCI.4408-05.2006 16407540 PMC6674402

[B61] Smith Y. Surmeier D. J. Redgrave P. Kimura M. (2011). Thalamic contributions to basal ganglia-related behavioral switching and reinforcement. *J. Neurosci.* 31 16102–16106. 10.1523/JNEUROSCI.4634-11.2011 22072662 PMC3235411

[B62] Snodgrass J. G. Vanderwart M. (1980). A standardized set of 260 pictures: norms for name agreement, image agreement, familiarity, and visual complexity. *J. Exp. Psychol. Hum. Learn. Mem.* 6 174–215. 10.1037/0278-7393.6.2.174 7373248

[B63] Snyder P. J. Harris L. J. (1993). Handedness, sex, familial sinistrality effects on spatial tasks. *Cortex* 29 115–134. 10.1016/S0010-9452(13)80216-X 8472549

[B64] Song X.-W. Dong Z.-Y. Long X.-Y. Li S.-F. Zuo X.-N. Zhu C.-Z. (2011). REST: a toolkit for resting-state functional magnetic resonance imaging data processing. *PLoS One* 6:e25031. 10.1371/journal.pone.0025031 21949842 PMC3176805

[B65] Sörbom D. (1989). Model modification. *Psychometrika* 54 371–384. 10.1007/BF02294623

[B66] Squire L. R. Stark C. E. Clark R. E. (2004). The medial temporal lobe. *Annu. Rev. Neurosci.* 27 279–306. 10.1146/annurev.neuro.27.070203.144130 15217334

[B67] Sridharan D. Levitin D. J. Menon V. (2008). A critical role for the right fronto-insular cortex in switching between central-executive and default-mode networks. *Proc. Natl. Acad. Sci. U.S.A.* 105 12569–12574. 10.1073/pnas.0800005105 18723676 PMC2527952

[B68] Stevens M. C. Kiehl K. A. Pearlson G. D. Calhoun V. D. (2007). Functional neural networks underlying response inhibition in adolescents and adults. *Behav. Brain Res.* 181 12–22. 10.1016/j.bbr.2007.03.023 17467816 PMC2266817

[B69] Tang C. van Heuven V. J. (2009). Mutual intelligibility of Chinese dialects experimentally tested. *Lingua* 119 709–732. 10.1016/j.lingua.2008.10.001

[B70] Thierry G. Wu Y. J. (2007). Brain potentials reveal unconscious translation during foreign language comprehension. *Proc. Natl. Acad. Sci. U.S.A.* 104 12530–12535. 10.1073/pnas.0609927104 17630288 PMC1941503

[B71] Tu L. Wang J. Abutalebi J. Jiang B. Pan X. Li M. (2015). Language exposure induced neuroplasticity in the bilingual brain: a follow-up fMRI study. *Cortex* 64 8–19. 10.1016/j.cortex.2014.09.019 25461703

[B72] Ventura-Campos N. Sanjuán A. González J. Palomar-García M.-Á. Rodríguez-Pujadas A. Sebastián-Gallés N. (2013). Spontaneous brain activity predicts learning ability of foreign sounds. *J. Neurosci.* 33 9295–9305. 10.1523/JNEUROSCI.4655-12.2013 23719798 PMC6618579

[B73] Wechsler D. (1997). *Wechsler Adult Intelligence Scale-(WAIS-III).* San Antonio, TX: Psychological Corporation.

[B74] Weissberger G. Gollan T. Bondi M. Clark L. Wierenga C. (2015). Language and task switching in the bilingual brain: bilinguals are staying, not switching, experts. *Neuropsychologia* 66 193–203. 10.1016/j.neuropsychologia.2014.10.037 25446970 PMC4596720

[B75] Woo C.-W. Krishnan A. Wager T. D. (2014). Cluster-extent based thresholding in fMRI analyses: pitfalls and recommendations. *Neuroimage* 91 412–419. 10.1016/j.neuroimage.2013.12.058 24412399 PMC4214144

[B76] Wu Y. J. Thierry G. (2010). Chinese–English bilinguals reading English hear Chinese. *J. Neurosci.* 30 7646–7651. 10.1523/JNEUROSCI.1602-10.2010 20519539 PMC6632379

[B77] Wu Y. J. Thierry G. (2013). Fast modulation of executive function by language context in bilinguals. *J. Neurosci.* 33 13533–13537. 10.1523/JNEUROSCI.4760-12.2013 23946411 PMC3742936

[B78] Wu Y. J. Thierry G. (2017). Brain potentials predict language selection before speech onset in bilinguals. *Brain Lang.* 171 23–30. 10.1016/j.bandl.2017.04.002 28445784

[B79] Yan C. G. Wang X. D. Zuo X. N. Zang Y. F. (2016). DPABI: data processing & analysis for (resting-state) brain imaging. *Neuroinformatics* 14 339–351. 10.1007/s12021-016-9299-4 27075850

[B80] Yang J. Gates K. M. Molenaar P. Li P. (2015). Neural changes underlying successful second language word learning: an fMRI study. *J. Neurolinguistics* 33 29–49. 10.1016/j.jneuroling.2014.09.004

[B81] Yang J. Li P. (2012). Brain networks of explicit and implicit learning. *PLoS One* 7:e42993. 10.1371/journal.pone.0042993 22952624 PMC3432050

[B82] Zou L. Abutalebi J. Zinszer B. Yan X. Shu H. Peng D. (2012). Second language experience modulates functional brain network for the native language production in bimodal bilinguals. *Neuroimage* 62 1367–1375. 10.1016/j.neuroimage.2012.05.062 22658973

