# Supplementary material for: Bilingual Contexts Modulate the Inhibitory Control Network
Source: Front Psychol. 2018 Mar 27;9:395. doi: 10.3389/fpsyg.2018.00395 (PMC5881103; doi:10.3389/fpsyg.2018.00395)
Supplement: Supplementary file 1 [file Data_Sheet_1.DOCX]

Supplementary Materials for

**Bilingual Contexts Modulate the Inhibitory Control Network**

Jing Yang, Jianqiao Ye, Ruiming Wang, Ke Zhou, and Yan Jing Wu

This PDF file includes: Figure S1. Brain activations for switching effects in L1-L2 and L2-L3 picture naming tasks.


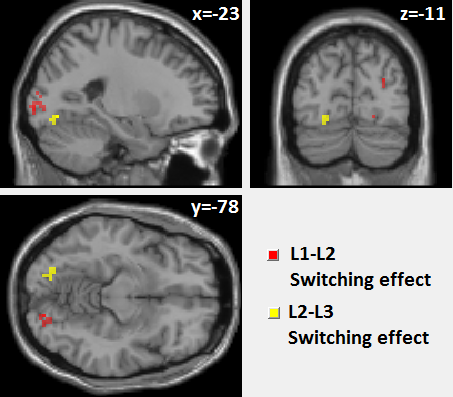


**Figure S1. Brain activations associated with switching effects in the L1-L2 and the L2-L3 blocks.**

When participants named pictures in the L1-L2 block, switching trials (in both L1 and L2), as compared to non-switching trials, showed higher neural activities in the bilateral superior, middle occipital lobes, and the right superior parietal lobule (**Red**). In the L2-L3 block, the same contrast resulted in more brain activations in the left fusiform gyrus (**Yellow**). No significant brain activation was found for language switches in the L1-L3 block.
